# Self-Propelled Janus Microdimer Swimmers under a Rotating Magnetic Field

**DOI:** 10.3390/nano9121672

**Published:** 2019-11-22

**Authors:** Shimin Yu, Ningze Ma, Hao Yu, Haoran Sun, Xiaocong Chang, Zhiguang Wu, Jiaxuan Deng, Shuqi Zhao, Wuyi Wang, Guangyu Zhang, Weiwei Zhang, Qingsong Zhao, Tianlong Li

**Affiliations:** 1 State Key Laboratory of Robotics and System, Harbin Institute of Technology, Harbin 150001, China; qdyushimin@163.com (S.Y.); Mnz333@126.com (N.M.); yu1997hao@gmail.com (H.Y.); 17390600488@163.com (H.S.); xiaocongchang@hotmail.com (X.C.); zhiguangwu@hit.edu.cn (Z.W.); 1170801208@stu.hit.edu.cn (J.D.); wangwuyi@hit.edu.cn (W.W.); zgyqx@hit.edu.cn (G.Z.); 2 Institute of Pharmacy, Sechenov University, 119991 Moscow, Russia; 3 College of Control Science and Engineering, Zhejiang University, Hangzhou 310058, China; shuqi010421@163.com; 4 School of Mechanical Engineering, Zhengzhou University, Zhengzhou 450001, China; 5 Department of endocrinology, Harbin Medical University, Harbin 150001, China

**Keywords:** Janus microdimer, propulsion mechanism, rotating magnetic field

## Abstract

Recent strides in micro- and nanofabrication technology have enabled researchers to design and develop new micro- and nanorobots for biomedicine and environmental monitoring. Due to its non-invasive remote actuation and convenient navigation abilities, magnetic propulsion has been widely used in micro- and nanoscale robotic systems. In this article, a highly efficient Janus microdimer swimmer propelled by a rotating uniform magnetic field was investigated experimentally and numerically. The velocity of the Janus microdimer swimmer can be modulated by adjusting the magnetic field frequency with a maximum speed of 133 μm·s^−1^ (≈13.3 body length s^−1^) at the frequency of 32 Hz. Fast and accurate navigation of these Janus microdimer swimmers in complex environments and near obstacles was also demonstrated. This efficient propulsion behavior of the new Janus microdimer swimmer holds considerable promise for diverse future practical applications ranging from nanoscale manipulation and assembly to nanomedicine.

## 1. Introduction

Micro-/nanoswimmers that can convert various types of energy into kinetic energy overcoming viscous drag forces and thermal fluctuations have demonstrated different tasks in various environments [1,2,3,4,5,6,7,8,9]. Recent strides in nanotechnology have enabled researchers to develop micro- and nanorobot systems to perform great potential in the fields of drug delivery [10,11,12,13,14,15], biosensing [16,17], self-assembly [18,19,20], micro-manipulation [21,22,23], environmental detection and remediation [24,25,26,27,28] and super-resolution optical imaging [29,30]. However, rapid and accurate motion in low Reynolds number environments requires new specialized techniques. Therefore, it is still of particular importance to develop micro- and nanorobot by easy fabrication, capable of efficient propulsion and accurate navigation.

Unicellular living organisms have a distinctive ability to locomote efficiently by non-reciprocal motion mechanism in different environments [31]. Inspired by the swimming strategies of natural microorganisms, various functional micro-/nanorobots, which are propelled by several external excitations (e.g. chemistry [32,33,34,35,36], light [37,38,39,40,41,42], magnetic [43,44,45,46,47,48,49,50,51], ultrasonic [13,52,53,54] and electric [55]) have been developed in this decade. Among these propulsion methods, magnetic propulsion has been widely used to power the micro- and nanorobots due to its non-invasive remote actuation and convenient navigation abilities [56,57,58]. According to propulsion mechanisms, the magnetic propelled micro-/nanorobots can be further categorized into two groups: rotating magnetic field propulsion and oscillating magnetic field propulsion. Inspired by helical bacterial flagella, the first group of micro-/nanorobots locomotes upon rotation induced by rotating magnetic fields [59]. For example, Nelson’s group designed and fabricated a variety of magnetic helical micromachines that could achieve self-propulsion, controllable collective behavior, self-assembly and cargo transport in magnetic fields [43,59,60]. The helical microswimmers with different surface wettability properties were fabricated to achieve selective control of individual swimmers with different speed, and the maximum velocity up to 62 µm·s^−1^ (≈4.5 body length s^−1^) [61]. Another type of micro-/nanorobots, as inspired by the oscillating propulsion of sperm, relays on the asymmetrical shape deformation to break the spatial symmetry and escape the constraints from scallop theorem [31,62]. For instance, Fischer’s group investigated the reciprocal motion of symmetric ‘micro-scallop’ microswimmer at low Reynolds numbers, which can propel microswimmer in shear thickening and shear thinning (non-Newtonian) fluids [63]. In addition, Li fabricated a new type of magnetic surface walker that can achieve speeds of up to 18.6 µm·s^−1^ (≈4.0 body length s^−1^) in an oscillating magnetic field [44]. However, efficient propulsion gaits at the nanoscale are still quite limited. In particular, faster microswimmers and correspondent drive systems need to be developed for a wide range of proposed applications.

Here, we report a highly efficient Janus microdimer swimmers propelled by a rotating uniform magnetic field. This Janus microdimer swimmer consists of two Ni/SiO_2_ Janus microsphere connected by magnetic forces. The microdimer swimmer, which relies on a surface to escape the constraints from the scallop theorem, can rotate efficiently in low Reynold fluids in response to an external magnetic field. It is capable of a powerful propulsion up to 133 μm·s^−1^ (≈13.3 body length s^−1^) at a driving frequency of 32 Hz and a magnetic field strength of 5 mT. Furthermore, autonomous navigation of swimmers in complex environments was also demonstrated. This new Janus microdimer swimmers can open new possibilities for biomedical operation at the nanoscale.

## 2. Materials and Methods

### 2.1. Rotating Uniform Magnetic Field Setup

**Figure 1:**
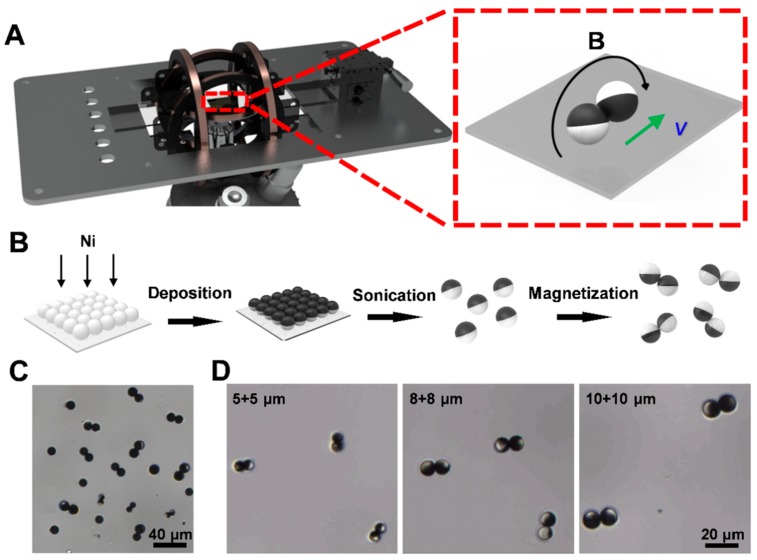
Design and fabrication of Janus microdimer swimmers. (**A**) schematic of rotating magnetic field generation system; (**B**) fabrication of Janus microdimers; (**C**) optical microscopy image of microdimers after magnetization; (**D**) representative microdimers of different sizes.

Figure 1A displays the setup of an external rotating uniform magnetic field, which consists of a three degrees of freedom Helmholtz coil, a multifunction data acquisition and a three single-channel output power amplifier. Based on controlling the current and the voltage of Helmholtz coil, an external rotating uniform magnetic field can be circularly generated in any plane of 3D space to actuate the microrobots in different motion modes. In order to achieve real-time observation of swimmers, the external magnetic field setup was placed on the observation platform of the microscope to achieve real-time observation of swimmers.

### 2.2. Fabrication of the Janus Microdimer

The preparation process of the Janus microdimer is shown in Figure 1B. The 5, 8 and 10 μm silica microspheres were first washed three times with deionized (DI) water. Then the SiO_2_ microspheres were placed onto glass slides and deposited by an ion-sputtering apparatus (K575XD, Emitech, Laughton, England) at 90° angle of incidence to be coated with a 100 nm nickel layer [12,64]. Different thickness and coverage area of the Ni layer can be obtained by changing deposition time and the angle of incidence. After a brief sonication in ultrapure water, the Janus microspheres were released from the glass slide and dispersed into ultrapure water. The Janus microspheres were stored in ultrapure water until use. 

As they are exposed to an external magnetic field, the SiO_2_-Ni Janus microspheres were attracted to each other because of the magnetic polarization of the Ni layer (Figure 1B), and the attachment can be changed by controlling the thickness and coverage area of Ni layer on microspheres. Figure 1C,D showed the microdimers consist of two Janus microspheres with same diameter. These microdimers can move along specific direction in an external rotating uniform magnetic field, which will be discussed in more detail later. 

### 2.3. Optical Observation and Tracking

Videos of Janus microdimer swimmers were captured at 25 frame·s^−1^ by an inverted optical microscope (IX73, Olympus, Tokyo, Japan) coupled with a 20× objective and a Point Grey CCD camera (GS3-U3-51S5C/M-C, FLIR, Wilsonville, OH, United States). These video data were analyzed using ImageJ and MATLAB to obtain the trajectories and velocities of swimmers.

## 3. Results and Discussion

### 3.1. Propulsion of Microdimer Swimmers

**Figure 2:**
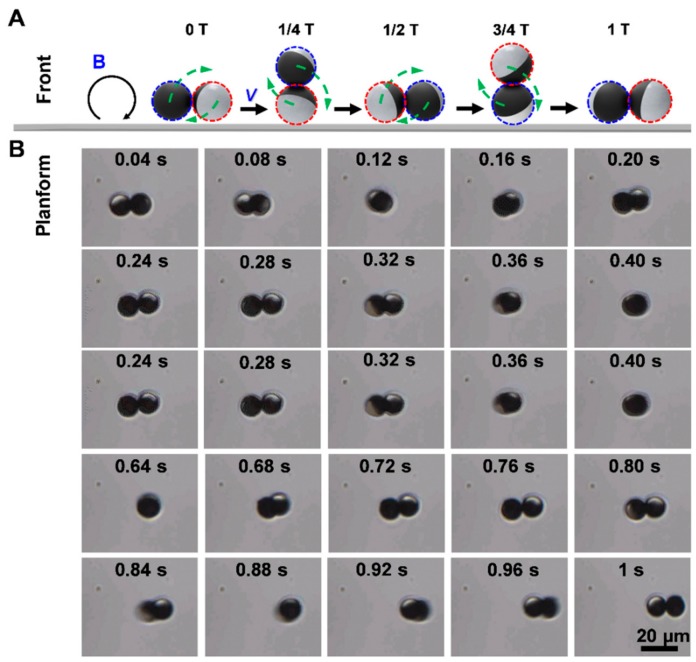
Propulsion of microdimer swimmer under a rotating uniform magnetic field. (**A**) propulsion mechanism of microdimer in a rotating magnetic field; (**B**) time-lapse optical microscopy images depicting the motion of a microdimer within ~1 s.

Reciprocal motion in low Reynolds number fluids hinders directional driving of symmetry miniaturized objects. In our experiments, the presence of the surface wall is a key factor for microdimer swimmers to escape the constraints from the time-reversible symmetry. Figure 2A shows the propulsion mechanism of microdimer swimmers in a rotating uniform magnetic field. When the magnetic field is applied, the microdimer swimmer is rolled by the magnetic torque. During the first half of the cycle, the blue sphere in the microdimer rolls forward and red one rolls backward. However, the viscous drag due to the proximity of the surface reduces the speed of the red sphere obviously, and causes the center of mass of the swimmer to move dominantly forward. During the next half of the cycle, the two spheres switch roles, and the rapid rolling of the red ball prompts a net displacement of the swimmer. The two spheres alternated back and forth to drive the microdimer swimmer movement along specific direction. This continuous motion process of 1 s (2 cycles) at a magnetic field strength of 5 mT and a driving frequency of 2 Hz was captured and is shown in Figure 2B (Video S1, Supplementary Materials). As can be seen, the two microspheres constituting the microdimer swimmer alternately rolled forward and propelled the swimmer ~24 μm (≈1.2 body length) in a straight line within 1 s. 

**Figure 3:**
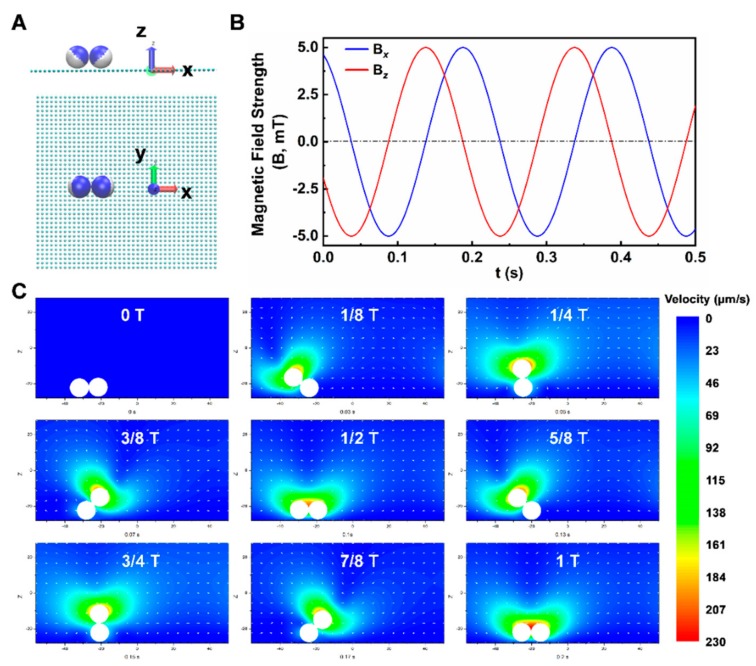
Simulation of microdimer swimmer under a rotating magnetic field and near-wall flow field. (**A**) side and top views of the simulation models. Janus microspheres are represented by the blue and white balls, and the wall surface is depicted in cyan; (**B**) the applied rotating magnetic field with frequency of 5 Hz and strength of 5 mT; (**C**) the sequence profile of near-wall flow field surrounding the microdimer swimmer.

Fluidic interaction is another critical factor for triggering the controllable propulsion of microdimer swimmers [65]. Janus particles will experience a drag force due to the difference of velocity between particles and surrounding fluids. The fluidic interaction can keep the microdimer swimmers in contact with the wall surface continuously, and transform the rotation movement of microdimer swimmers into a linear movement along the water/wall interface. As shown in Figure 3, the fluidic velocity field induced by a rotating microdimer swimmer adjacent to the wall surface has been simulated and analyzed. All the simulations were performed within the framework of a large-scale atomic/molecular massively parallel simulator (LAMMPS), which is a highly parallelized solver for molecular dynamics simulations [66]. The Lattice Boltzmann method (LBM), which is an efficient and accurate method for Newtonian flow [67], was employed to solve Navier–Stokes equations. The LBM solver was directly embedded into LAMMPS as a fix_lb_fluid [68], where fix is a kind of class offered by LAMMPS to apply external control on the simulation system. Each Janus microsphere was treated as a sphere with a point dipole shifted from the geometric center of microsphere (Figure 3A) [69]. The anisotropic magnetic susceptibility was scaled by the experimental hysteresis curve and the dipole–moment shift was determined by matching the experimentally observed bond angle of the zigzag chain in a static magnetic field. As shown in Figure 3B, magnetic interactions were determined at each time step by solving the linear system of equations for each microsphere’s magnetic moment as a function of the field produced by the other microspheres and the spatially uniform, time-dependent external field. The movement of a magnetic Janus microsphere was captured by solving Newton’s second law equation, under the influence of both hydrodynamic force and magnetic force (the field strength at 5mT and frequency at 5 Hz). 

The flow profile shows that the maximal magnitude of the flow field surrounded the rear microsphere throughout the first half of the motion period. This indicates the faster rotation of rear microsphere than the front one due to the wall effect. Then, the strong flow took place nearby the front microsphere by turn during the latter half of motion period, and the rear sphere was alternatively dragged close to the wall surface by the fluidic interaction and the gravity force of the microdimer. However, the near-wall sphere was not fixed on the wall. The flow profile in Figure 3C also exhibits week flow fields behind the near-wall sphere at 0.07 s and 0.17 s. This reveals that the near-wall microsphere just slid on the wall, which can be further confirmed by the net displacement of microdimer swimmer. After a complete rotation cycle, the Janus swimmer advanced about a half length of the microdimer as shown in Figure 3C, which should be one-body length with no-slip condition.

### 3.2. Analysis of the Motion Law of Microdimer Swimmers

**Figure 4:**
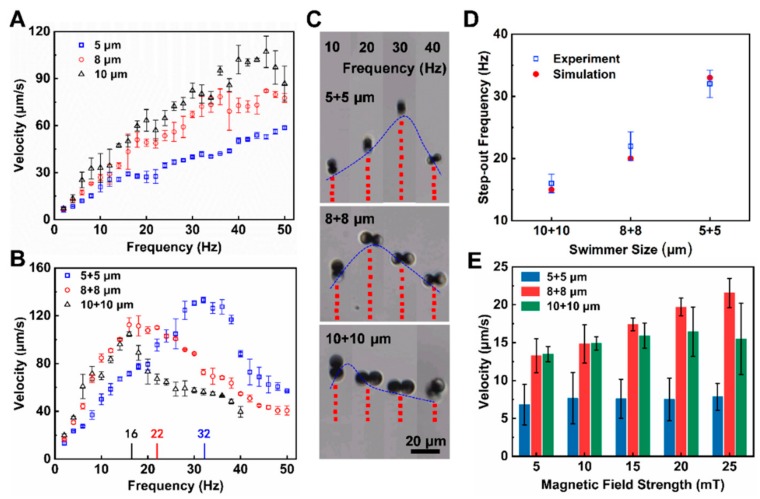
Performance of microdimer swimmers under different experimental parameters. The velocity of (**A**) single microspheres and (**B**) microdimers varied with the drive frequency; (**C**) tracking lines illustrating the traveled distances of different microdimers over a 1 s period in a rotating uniform magnetic field with frequencies from 10 to 40 Hz; (**D**) simulation results of microdimers velocity varied with the drive frequency; (**E**) velocity of microdimers at different magnetic field strength with a driving frequency of 1 Hz.

To investigate the principle behind microdimer motion, we turned our attention to their velocity under different magnetic field parameters, which is essential for the industrial and medical applications of microdimers [59]. First, the motion law of single Janus microsphere was investigated. When a single microsphere was exposed to a rotating magnetic field, the torque induced by rotating magnetic field and the viscous drag due to the proximity of the surface broke the reciprocal motion of single microsphere and caused it to roll forward along the surface. The dependence of the velocity of single Janus microsphere with different sizes on the driving frequency was characterized, as shown in Figure 4A. The velocity of the 5 μm Janus microsphere increased from 6.5 to 58.6 µm·s^−1^ (≈10.7 body length s^−1^) upon increasing the driving frequency from 2 to 50 Hz. The 8 and 10 µm Janus microspheres presented the similar speed trends, and their speeds increased to 82.2 µm·s^−1^ (≈10.3 body length s^−1^) and 107.1 µm·s^−1^ (≈10.7 body length s^−1^), respectively. This result illustrates that relative speed (body length s^−1^) of Janus microspheres is frequency-dependent and is constant over different sizes. Notably, the speeds of the larger Janus microspheres were higher than those of the smaller one under same driving frequency. These results show the linear relation between the velocity of Janus microsphere and driving frequency.

Then the effect of frequency of the rotating magnetic field on the velocity of microdimer swimmers was investigated experimentally as well. The driving frequency increased from 2 to 50 Hz with a magnetic field strength of 5 mT, as shown in Figure 4B. For a 5 + 5 μm microdimer swimmer, the speed increased linearly with the driving frequency and reached a maximum velocity of 133 μm·s^−1^ (≈13.3 body length s^−1^) at 32 Hz, further increasing the frequency reduced the velocity. Such a maximum synchronized frequency is called step-out frequency which was also commonly observed for many other types of micromotors in rotating and oscillating magnetic fields [44,70,71]. The reason we speculate for this variation is the occurrence of out-of-step phenomenon and the increase in drag caused by the increasing speed. Furthermore, the 8 + 8 and 10 + 10 μm microdimer swimmers obtained the highest velocities of 110 μm·s^−1^ (≈6.9 body length s^−1^) and 89 μm·s^−1^ (≈4.5 body length s^−1^) at 22 and 16 Hz, respectively. This result illustrates that the step-out frequencies of the larger microdimer swimmer are lower than those of the smaller microdimers under the same conditions, which is similar to the performance of microdimers in an oscillating magnetic field [44]. Figure 4C displays the trajectories of microdimer swimmers in different sizes at driving frequencies from 10 to 40 Hz over a period of 1 s. The microdimer swimmers continuously moved linearly aligning on rotation direction of the magnetic field, and higher speeds were achieved near the step-out frequency. In order to verify the variation law of this step-out frequency, the microdimer swimmer speed under different swimmer sizes and driving frequency was simulated and the step-out frequency was analyzed, as shown in Figure 4D. In the simulation, the step-out frequencies of the 5 + 5, 8 + 8, 10 + 10 μm microdimer swimmers were 33, 20 and 15 Hz, respectively, which was in good agreement with the experimental results. 

In addition to driving frequency, magnetic field strength is also an important parameter of external rotating magnetic field [72]. Hence, we further studied the effect of magnetic field strength on the performance of microdimer swimmers, as shown in Figure 4E. At driving frequency of 1 Hz, the velocity of 8 + 8 μm microdimer swimmer increased from only 13 to 21 μm·s^−1^ upon increasing magnetic field strength from 5 to 25 mT, while the velocities of the 5 + 5 and 10 + 10 μm microdimer swimmers were almost constant. It illustrates that under the current parameters, varying the magnetic field strength has little effect on the velocity of the microdimer swimmer compared to the driving frequency. We suspect that at such lower speed, the microdimer swimmers are subjected to a propulsion magnetic force much larger than the drag, so the increase in magnetic field strength do not effectively improve the rotational speed of the swimmer which directly determines the net displacement velocity of the swimmers.

### 3.3. Controllable and Flexible Motility Performance of Microdimer Swimmers 

**Figure 5:**
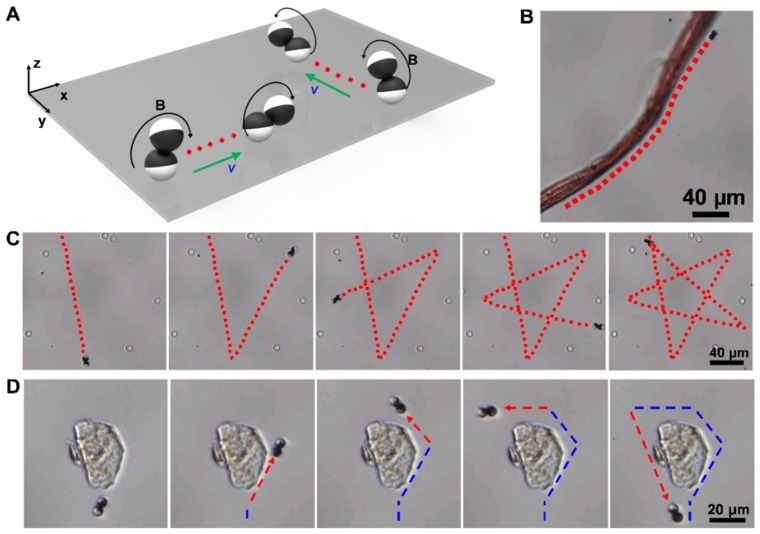
Controllable and flexible motility performance of microdimer swimmers. (**A**) change of the direction of movement of the microdimer swimmer caused by changing the magnetic field; (**B**) controllable curve motion of microdimer swimmer; (**C**) ‘star’ trajectory of microdimer swimmer; (**D**) how a microdimer swimmer detoured around an obstacle.

The abilities of remote actuation and to avoid obstacles are highly attractive features for micro- and nano-scale swimmers in the application of precision medical procedures [3,73]. Here, we demonstrate the remote navigation of Janus microdimer swimmers. Figure 5A illustrates the control strategy of three-dimensional rotating magnetic field generated by the three degrees of freedom Helmholtz coil. First, a circularly polarized rotating magnetic field given by *H*(t) = *H*_0_[cos(ωt)**e**_x_ + sin(ωt)**e**_z_] was applied in the *x*-*z* plane, the microdimer swimmer rolled along *x* axis. Here, *H*_0_ is the magnitude of *H*(t), ω is the angular frequency of the magnetic field, t is the time, and **e**_x_ and **e**_z_ are the unit vector along the *x* and *z* axes, respectively (hereafter, **e**_y_ is that along the *y* axis). When the rotating magnetic field was changed and applied in the *y*-*z* plane, given by *H*(t) = *H*_0_[−cos(ωt)**e**_y_ + sin(ωt)**e**_z_], the direction of microdimer swimmer motion changed to the *y* axis. The propulsion direction of the microrobot could be altered by changing the direction of rotating magnetic field, which could be achieved by control input current manually. First, Figure 5B shows the curved motion of a microdimer swimmer along the edge of a ribbon obstacle (Video S2, Supplementary Materials). The swimmer’s trajectory fitted well with the edge of the obstacle, which means that the motor’s direction of motion can be controlled continuously. Based on the above sensitive magnetic orientation of microdimer swimmers, a swimmer walked along a predefined star-shape trajectory in the gap of 8 μm non-magnetic microspheres, as shown in Figure 5C (Video S3, Supplementary Materials). The corners of the ‘star’ track line were achieved easily by changing the magnetic field angle by ~134°. Finally, we controlled a microdimer swimmer to detour around an obstacle that was much larger than their own volume and return to the original position as shown in Figure 5D (Video S4, Supplementary Materials). The swimmer walked the optimal path according to the outer contour of the large obstacle to bypass it.

## 4. Conclusions

In summary, we have demonstrated a new propulsion and steering system for a Janus microdimer swimmer under a rotating uniform magnetic field. A maximum speed of 133 μm·s^−1^, corresponding to a relative velocity of 13.3 body length s^−1^, was obtained by using a rotating uniform magnetic field with a frequency of 32 Hz and a magnetic strength of 5 mT. On-demand modulation of the speed was easily achieved by ramping the magnetic field strength and frequency up and down. Based on the transformable alignment of the two Janus spheres upon the rotating magnetic field, precise and remote navigation of microdimer swimmers provided good controllable ability of the locomotion trajectory and the ability to avoid obstacles. Due to its non-invasive remote actuation and convenient navigation, the efficient propulsion and steering system can open the door for a wide variety of applications ranging from nanomanipulation to precise medical treatment.

## Supplementary Materials

The following are available online at https://www.mdpi.com/2079-4991/9/12/1672/s1: Video S1: Near-wall motion of micro-dimer swimmer caused by rotating magnetic field, Video S2: Curved motion of a microdimer along the edge of a ribbon obstacle, Video S3: “Star” trajectory movement of microdimer swimmer, Video S4: Obstacle avoidance movement of microdimer swimmer.
